# Tratamento da recidiva de reestenose intra-stent renal por angioplastia com balão farmacológico

**DOI:** 10.1590/1677-5449.005117

**Published:** 2018

**Authors:** Rodrigo Gibin Jaldin, Marcone Lima Sobreira, Regina Moura, Matheus Bertanha, Rafael Elias Fares Pimenta, Ricardo de Alvarenga Yoshida, Jamil Victor de Oliveira Mariúba, Winston Bonetti Yoshida

**Affiliations:** 1 Universidade Estadual Paulista – UNESP, Faculdade de Medicina de Botucatu, Departamento de Cirurgia e Ortopedia, Botucatu, SP, Brasil.

**Keywords:** artéria renal, estenose arterial, hipertensão renovascular

## Abstract

Nos últimos anos, balões farmacológicos surgiram como promissora alternativa terapêutica em intervenções endovasculares. Com essa tecnologia, transferem-se drogas antiproliferativas à parede arterial, sem a necessidade de implante metálico para liberação. Descreve-se o caso de um paciente com uma segunda recidiva de reestenose intra-stent renal tratada por angioplastia com balão coberto por droga, com boa evolução clínica caracterizada por adequado controle pressórico e redução de classes e dosagem dos anti-hipertensivos. Os resultados obtidos com balões farmacológicos em outros territórios e esta experiência isolada podem contribuir como sugestão para o uso desses dispositivos na reestenose intra-stent renal, com resultados iniciais satisfatórios.

## INTRODUÇÃO

 A reestenose após angioplastia resulta da interação de processos mecânicos e biológicos iniciados após a insuflação do balão: *recoil* precoce, remodelamento negativo e proliferação neointimal 1 . Essa limitação foi amenizada pela introdução de stents metálicos, nos quais se observa patência primária de 75% em 6 meses na artéria renal contra 29% no tratamento apenas por angioplastia com balão 2 . Porém, as taxas de reestenoses intra-stent renal variam entre 0-40%, com média em torno de 17% na maioria dos estudos para a primeira reestenose 2 ^,^ 3 . Um possível tratamento para recidivas após a angioplastia com stent seria a introdução de novo stent por dentro do stent prévio, porém novas reestenoses podem ocorrer em 36-71,4% dos casos 4 . 

 Os stents farmacológicos (*drug-eluting stents –* DES) surgiram com a expectativa de melhores resultados em relação à estenose intra-stent, particularmente em artérias renais de pequeno calibre, porém requerem terapia antiagregante plaquetária de longa duração e manutenção da estrutura metálica carreadora da droga 5 ^,^ 6 . Os dados sobre o uso de DES no tratamento das reestenoses intra-stent são controversos 7 . Descreve-se que 71% das artérias renais com estenose intra-stent tratadas com DES coaxial desenvolveram reestenose 7 ^-^ 9 . 

 Recentemente, balões farmacológicos (*drug-coated balloons –* DCBs) surgiram como alternativa em intervenções endovasculares 10 ^,^ 11 . Com esses, transfere-se rapidamente drogas antiproliferativas à parede arterial, sem necessidade de implante de estrutura metálica. Os promissores resultados dos DCBs em estudos clínicos nos diversos territórios arteriais poderiam sustentar sua aplicação nas artérias renais 11 . O uso desse tipo de dispositivo em reestenose intra-stent renal não está contemplado nas recomendações do fabricante e foi encontrado como opção terapêutica em um único relato de caso 12 . 

### Parte I – Situação clínica

**Figura 1:**
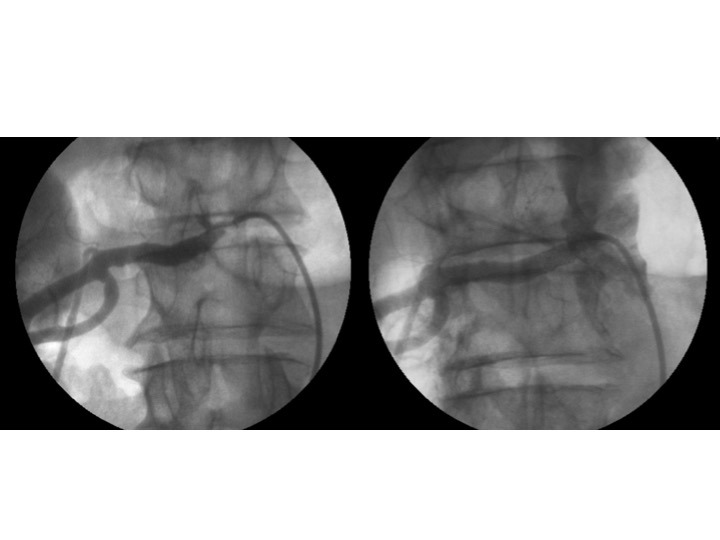
Primeira reestenose intra-stent renal. Angiografias pré e pós-reintervenção, sendo realizado implante de stent coaxial de 7 × 19 mm.

 Paciente masculino, 68 anos, branco, com antecedente de coronariopatia em pós-operatório tardio de revascularização do miocárdio, exclusão funcional crônica do rim esquerdo de provável causa aterosclerótica (confirmada por cintilografia) e estenose > 70% em arteriografia da artéria renal direita, tratada inicialmente pelo Serviço de Cardiologia Intervencionista com implante de stent balão-expansível de cromo-cobalto de 5 × 15 mm. Passados 7 anos, retornou com piora súbita dos níveis séricos de potássio (de 4,6 mg/dL para 6 mg/dL) e de creatinina (de 1,6 mg/dL para 10,4 mg/dL) e uremia (Ur = 230 mg/dL), com necessidade de hemodiálise temporária por 15 dias. Foi diagnosticada reestenose intra-stent > 80% por angiografia seletiva como causa da descompensação clínica. Foi tratado novamente pela Cardiologia Intervencionista com implante de stent balão-expansível de cromo-cobalto 7 × 19 mm no interior do stent prévio ( Figura 1 ), com melhora progressiva dos níveis de creatinina, que estabilizaram após 45 dias em torno de 1,4 mg/dL. 

 Após 3 meses, foi encaminhado para o Serviço de Cirurgia Vascular e Endovascular da Faculdade de Medicina de Botucatu para correção endovascular do aneurisma de aorta abdominal infrarrenal, que ocorreu sem intercorrências e sem elevação significativa dos níveis de creatinina após 72h do procedimento. Observou-se reestenose intra-stent renal de 60% na aortografia intraoperatória. Fazia uso regular de três classes de anti-hipertensivos e uso esporádico de clonidina, mantendo pressão sistólica média de 150 mmHg, segundo aferições diárias na unidade de saúde. Durante o seguimento, o mapeamento dúplex de aorta evidenciou endoprótese aórtica pérvia, sem vazamentos, e apontou reestenose > 70% intra-stent na artéria renal direita (velocidade de pico sistólico – VPS = 475 cm/s e índice renal-aórtico – IRA = 5,8). Diante desse quadro, algumas opções terapêuticas foram discutidas:

1 - Autotransplante renal
2 - Angioplastia com balão convencional
3 - Angioplastia com implante de um novo stent intra-stent
4 - Angioplastia com *cutting balloon*
5 - Angioplastia com implante de DES
6 - Angioplastia com balão farmacológico (DCB)

### Parte II - O que foi feito

**Figura 2:**
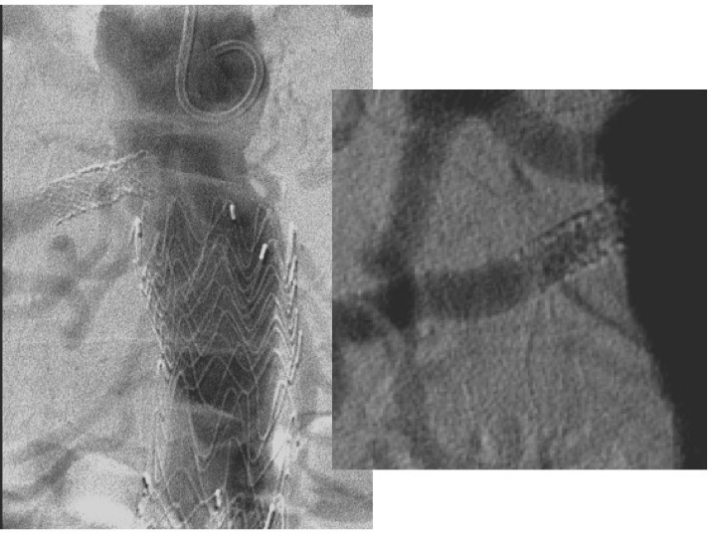
Aortografia por acesso braquial evidenciando sinais de hiperplasia intra-stent que confirmam o achado do mapeamento dúplex.

**Figura 3:**
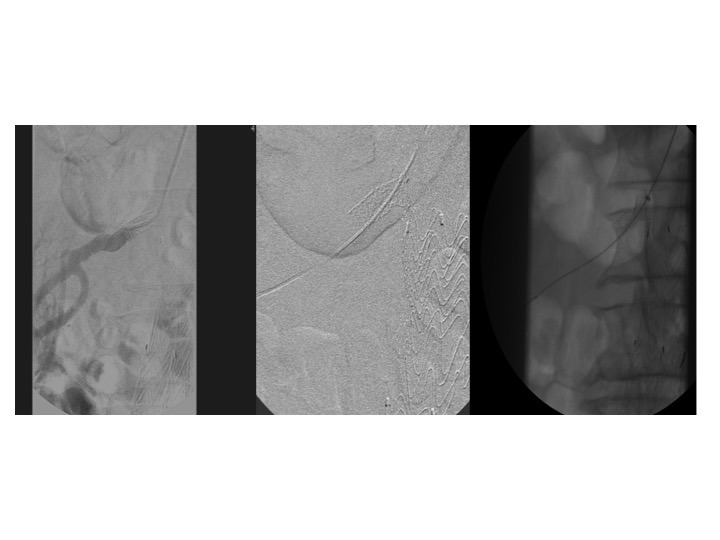
Sequência de acesso para a angioplastia intra-stent com balão com droga. Cateterismo seletivo da renal direita com cateter vertebral 5F 125 cm, transposição da lesão com guia hidrofílico *stiff* 0,035” 260 cm, troca de guia por extra-*stiff* 0,035” 260 cm e progressão de introdutor 6F 90 cm até a emergência da renal direita.

**Figura 4:**
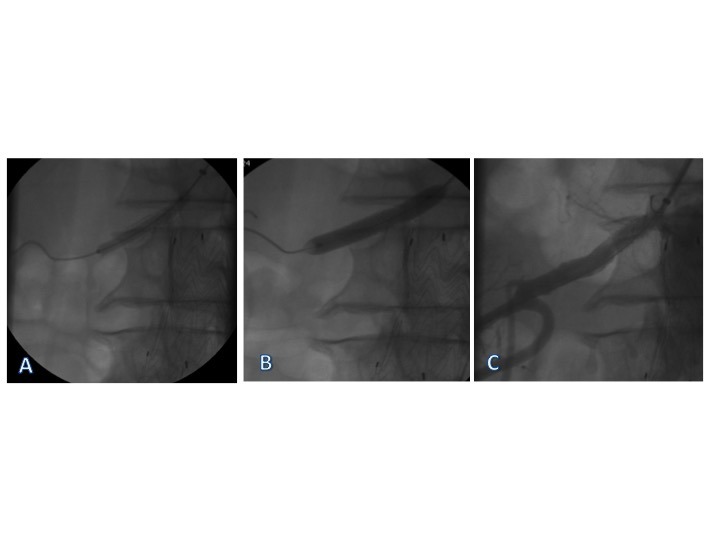
Pré-dilatação da lesão com balão 4 × 40 mm (A) e angioplastia com balão 6 × 40 mm coberto por paclitaxel, com evidência de “cintura” no balão no ponto de maior estenose seguida por expansão completa do balão até sua pressão nominal (B). Angiografia seletiva de controle pós-angioplastia mostrando estenose residual entre 30-40% (C).

 Indicou-se arteriografia renal e tratamento com balão farmacológico. Realizou-se acesso pela artéria braquial esquerda e aortografia, identificando-se estenose intra-stent de 70% ( Figura 2 ). Foi feito cateterismo seletivo da renal direita com cateter vertebral 5F 125 cm, transposição da lesão com guia hidrofílico *stiff* 0,035” 260 cm, troca de guia por extra-*stiff* 0,035” 260 cm e progressão de introdutor 6F 90 cm (Flexor – COOK®, Bloomington, USA), posicionado nas proximidades da emergência da renal direita ( Figura 3 ). Realizou-se pré-dilatação da lesão com balão 4 × 40 mm (Admiral Xtreme – Medtronic®, Minneapolis, USA), seguida de angioplastia com balão 6 × 40 mm coberto por paclitaxel (Admiral In.pact – Medtronic®, Minneapolis, USA), mantido insuflado na pressão nominal por 90 segundos. O resultado imediato foi estenose residual de 30%, uma vez que se manteve “cintura” central no balão, sem *recoil* significativo ou outros problemas associados, com utilização de aproximadamente 40 mL de contraste iodado não iônico de baixa osmolaridade ( Figura 4 ). 

**Figura 5:**
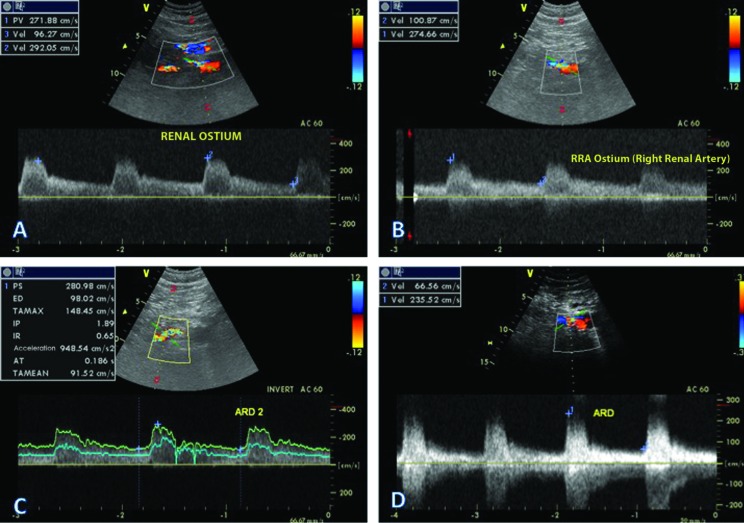
Avaliação periódica através do mapeamento dúplex renal. (A) Exame de 30 dias pós-angioplastia; (B) Exame de 90 dias pós-procedimento; (C) Exame de 6 meses pós-procedimento; (D) Exame de 12 meses pós-procedimento.

 Após 10 meses, o paciente apresentou melhora clínica, controle pressórico confirmado por MAPA, pressão sistólica média de 130 mmHg, redução de classes e dose dos anti-hipertensivos (manteve-se apenas enalapril 20 mg), sem qualquer alteração dos níveis de creatinina e ureia ou do *clearance* de creatinina. No seguimento até 24 meses, manteve melhora clínica e todos os exames mensais de mapeamento dúplex com critérios de estenose intra-stent entre 50-70% ( Figuras 5 e 6 ), segundo os critérios descritos por Chi et al. (VPS entre 225 e 315 cm/s e IRA entre 3,12 e 4,66) 13 até 2014 ( Figura 7 ). 

**Figura 6:**
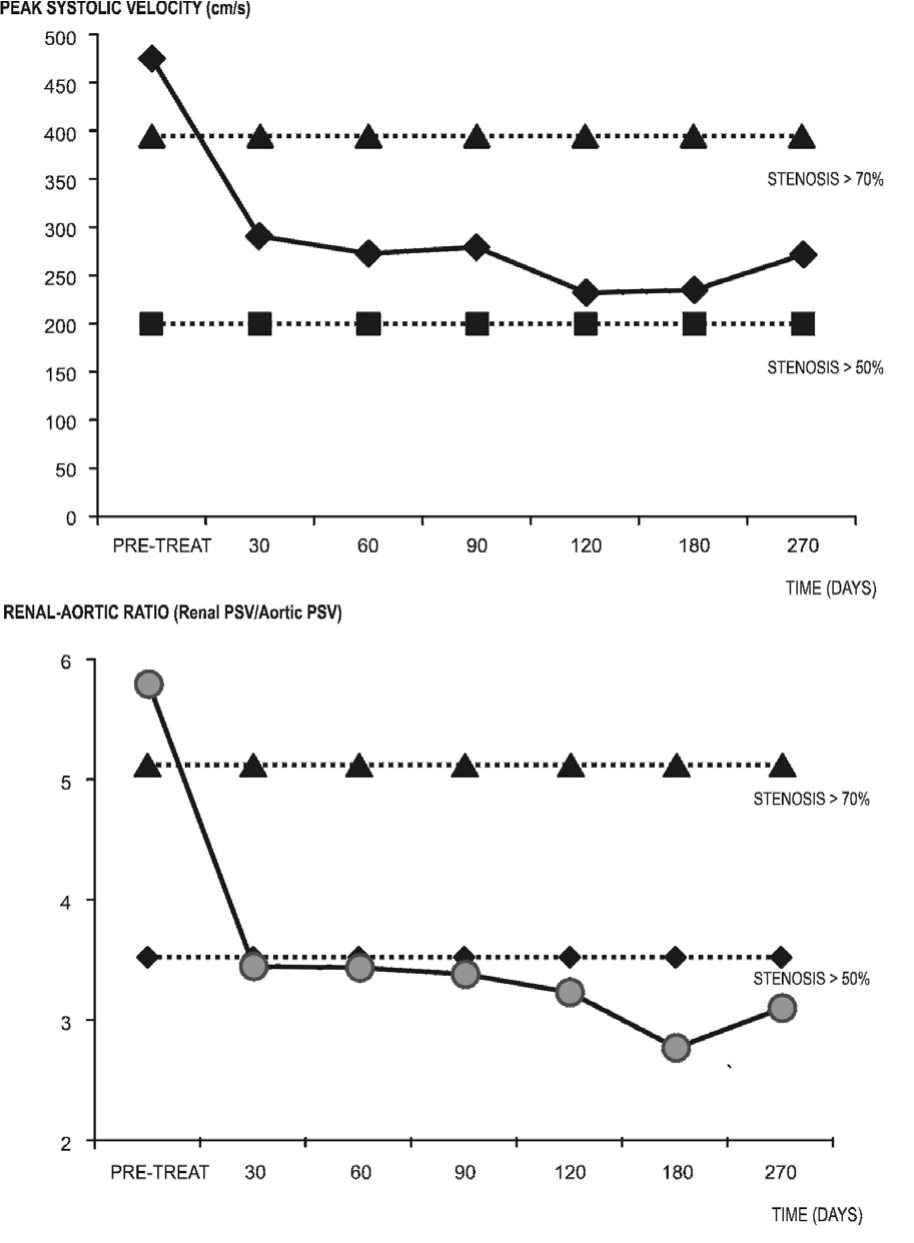
Curvas dos parâmetros Dopplervelocimétricos durante os primeiros 9 meses de seguimento pós-angioplastia. As linhas pontilhadas delimitam a faixa de estenose Dopplervelocimétrica entre 50-70%. Acima, velocidade de pico sistólico (VPS) em centímetros por segundo (cm/s). Abaixo, índice renal-aórtico (IRA), obtido pela relação entre as VPS medidas na artéria renal e na aorta suprarrenal.

**Figura 7:**
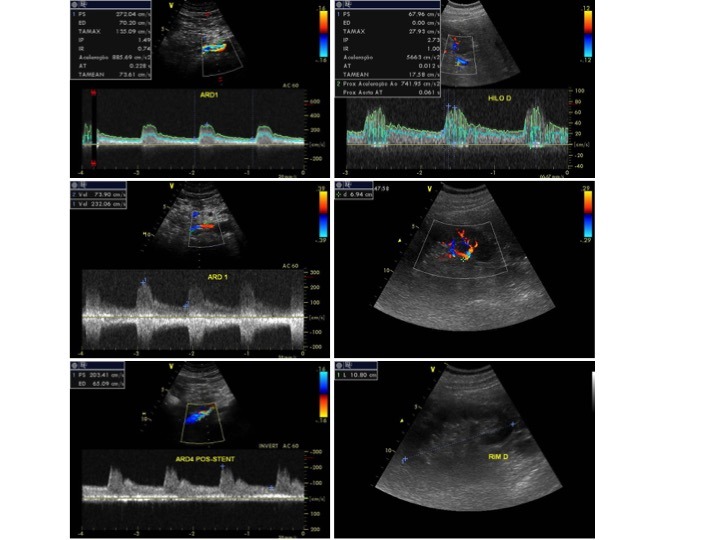
Mapeamento dúplex de artéria renal, hilar e parenquimatoso de controle de 24 meses pós-angioplastia com *drug-coated balloon* (DCB).

## DISCUSSÃO

 A estenose aterosclerótica da artéria renal ocorre em 1-5% da população hipertensa 14 ^,^ 15 e possui associação com o aneurisma de aorta abdominal em 20-38% dos casos 15 . Apesar do apelo inicial ao tratamento invasivo das lesões ateroscleróticas das artérias renais, atualmente tem se questionado seus reais benefícios quanto à prevenção da progressão da doença renal crônica e do real controle dos níveis pressóricos 16 ^-^ 19 . No caso apresentado, existia indicação para a angioplastia por tratar-se de revascularização renal por doença aterosclerótica em rim único 20 . 

 A reestenose após angioplastia é uma limitação da técnica percutânea de revascularização, independentemente do uso ou não de stents 16 ^-^ 21 . Em metanálise, a taxa de reestenose foi de 26% após angioplastia com balão e de 17% após angioplastia com stent 3 , o que respalda o uso de stent primário (classe I) para as estenoses ostiais da artéria renal, quando respeitadas as indicações para a intervenção endovascular. As taxas de reestenose após angioplastia renal com stent com resultado inicial satisfatório variam de 6-40%, dependendo do diâmetro do vaso tratado, das características da lesão e das comorbidades do paciente 2 ^,^ 3 ^,^ 8 . Outra possibilidade para a progressão da estenose intra-stent renal no caso exposto seria a correção endovascular do aneurisma de aorta a qual fora submetido 14 ^,^ 20 . Porém, seriam os dispositivos com fixação transrenal que estariam relacionados à progressão da estenose. No caso apresentado, a endoprótese utilizada para a correção do aneurisma se baseia em fixação infrarrenal. 

 A reestenose intra-stent de artéria renal de um rim único que mantenha sua função é uma grave complicação das angioplastias renais e pode ser prontamente tratada 12 . Não há consenso sobre o melhor tratamento para a reestenose intra-stent renal, mas a angioplastia com balão deve ser tentada inicialmente 8 . Descreve-se o uso de *cutting balloons*, crioplastia e implante de um novo stent dentro do anterior, mesmo não havendo boas evidências de uso e não sendo opções duráveis para o tratamento dessas lesões. Também se descreve a utilização de stents farmacológicos 8 ^,^ 22 ^,^ 23 ; porém, seu uso no tratamento das reestenoses intra-stent fornece resultados controversos e a própria sobreposição da malha metálica estimularia a proliferação neointimal excessiva 5 ^-^ 7 . 

 Nesse contexto, aparecem como alternativa interessante os balões farmacológicos. Com essa tecnologia, obtém-se a transferência de drogas antiproliferativas para a parede arterial em curto espaço de tempo, sem a necessidade de implante de sistema carreador 10 . O fato de a droga ser rapidamente colocada em contato com a superfície endotelial permitiria cicatrização vascular mais rápida e redução do processo inflamatório local. A familiaridade do cirurgião com os balões de angioplastia e a atual disponibilidade de drogas antiproliferativas clinicamente seguras e eficazes permitem seu uso seguro em diversos territórios arteriais 10 . 

 O agente antiproliferativo com maior número de estudos associados à tecnologia dos balões farmacológicos é o paclitaxel. Essa droga, oriunda da casca de uma árvore nativa do Pacífico (*Taxus brevifolia*), possui rápida absorção através da membrana celular por ser altamente lipofílica e atua através de ligação com a subunidade beta da tubulina, resultando em inibição da função dos microtúbulos. Isso causa modificação estrutural do citoesqueleto das células musculares lisas, alterando a proliferação e migração celular por aproximadamente 14 dias, sem apresentar citotoxicidade ou efeito rebote 10 ^,^ 24 . Em pequenos ensaios clínicos randomizados, os balões cobertos com paclitaxel reduziram as taxas de reestenose em pacientes com estenose intra-stent coronariano e em lesões femoropoplíteas 24 ^,^ 25 . 

 Apesar dos resultados satisfatórios dos DCBs em diversos territórios, restam dúvidas quanto ao seu uso de forma ampla. Não se sabe ao certo se a técnica é aplicável para o tratamento de superfícies manipuladas previamente, como áreas de endarterectomia ou intra-stent e não está elucidada a possibilidade de distribuição distal da droga e o impacto dessa situação em território visceral 12 . Entretanto, as baixas taxas de complicações relacionadas aos procedimentos sugerem que possam ser empregados na intervenção cardiovascular, com potencial aplicação nas artérias renais, fístulas de hemodiálise, território venoso e até valvoplastias percutâneas 9 ^,^ 10 . 

 No caso descrito, frente às inúmeras manipulações da artéria renal, à necessidade de intervenção e à possibilidade de aplicação dos balões cobertos por droga nas artérias renais e em superfície intra-stent, consideramos que o uso de DEB poderia ser uma alternativa pouco invasiva para o tratamento de uma situação complexa. Em seguimento de curto prazo, o paciente beneficiou-se clinicamente e em qualidade de vida com a terapêutica adotada. Os resultados obtidos com DCBs em outros territórios e esta experiência isolada pode contribuir como sugestão para o uso desses dispositivos na reestenose intra-stent renal. 
