# Supplementary material for: Tratamento da recidiva de reestenose intra-stent renal por angioplastia com balão farmacológico
Source: J Vasc Bras. 2018 Jan-Mar;17(1):81–8. [Article in Portuguese] doi: 10.1590/1677-5449.005117 (PMC5990271; doi:10.1590/1677-5449.005117)
Supplement: Translation

# Translation: Drug-coated balloon used to treat in-stent restenosis of the renal artery

**Authors:** Rodrigo Gibin Jaldin, Marcone Lima Sobreira, Regina Moura, Matheus Bertanha, Rafael Elias Fares Pimenta, Ricardo de Alvarenga Yoshida, Jamil Victor de Oliveira Mariúba, Winston Bonetti Yoshida

**Affiliations:** 1 Universidade Estadual Paulista – UNESP, Faculdade de Medicina de Botucatu, Departamento de Cirurgia e Ortopedia, Botucatu, SP, Brasil.

**Keywords:** in-stent restenosis, renal artery stenosis, renovascular hypertension

## Abstract

During recent years, drug-coated balloons (DCBs) have emerged as a promising therapeutic option. DCBs directly transfer antiproliferative drugs to the arterial wall in order to decrease myointimal hyperplasia. We describe a case of de novo renal artery in-stent restenosis (ISR) treated with drug-coated balloon angioplasty with acceptable short-term results, achieving blood pressure control using fewer antihypertensive agents. The experience and results obtained with DCBs in other territories could suggest and justify use of this technology in renal artery ISR.

## INTRODUCTION

 Restenosis after angioplasty is the result of interaction between mechanical and biological processes that are initiated after inflation of the balloon: early recoil, negative remodeling, and neointimal proliferation. 1 This limitation was ameliorated by the introduction of metal stents, with which primary renal artery patency rates of 75% at 6 months are observed, compared to 29% after treatment with balloon angioplasty alone. 2 However, rates of renal in-stent restenosis are in the range 0-40%, with a mean first restenosis rate of around 17% in the majority of studies. 2 ^,^ 3 One possible treatment for relapses after stent angioplasty is to insert another stent inside the original stent, but repeat restenoses can occur in 36-71.4% of cases. 4

 The development of drug-eluting stents (DES) offered hope of improved results in terms of in-stent stenosis, particularly in small caliber renal arteries, but they require long-duration anti-platelet treatment and maintenance of the metallic structure used to convey the drug. 5 ^,^ 6 Data on use of DES for treatment of in-stent restenoses are conflicting. 7 It has been reported that 71% of renal arteries with in-stent stenosis treated with coaxial DES develop restenosis. 7 ^-^ 9

 Recently, drug-coated balloons (DCBs) have emerged as an alternative option for endovascular interventions. 10 ^,^ 11 They rapidly transfer antiproliferative drugs to the artery wall, without the need to implant a metallic structure. The promising results of DCBs in clinical studies of a range of arterial territories may support their application in the renal arteries. 11 Use of this type of device for renal in-stent restenosis is not included in the manufacturers’ recommendations and has been described as a treatment option in a single case report. 12

### Part I – Clinical situation

 The patient was a 68-year-old white male with a history of coronary disease and was in the late postoperative period of myocardial revascularization, with chronic functional exclusion of the left kidney of probable atherosclerotic cause (confirmed by scintigraphy) and > 70% stenosis of the right renal artery, seen on arteriography, that had initially been treated with placement of a 5 × 15 mm chrome-cobalt balloon-expandable stent. He re-presented 7 years later with sudden increases in serum potassium (from 4.6 mg/dL to 6 mg/dL) and serum creatinine (from 1.6 mg/dL to 10.4 mg/dL) and uremia (Ur = 230 mg/dL), requiring temporary hemodialysis for 15 days. The cause of clinical decompensation was diagnosed by selective angiography as > 80% in-stent restenosis. He was once more treated by the Interventional Cardiology Service with placement of a 7 × 19 mm chrome-cobalt balloon-expandable stent inside the original stent ( Figure 1 ), with progressive improvement in creatinine levels, which stabilized at around 1.4 mg/dL after 45 days. 

**Figure 1:** First renal in-stent restenosis. Angiographs before and after the reintervention, in which a 7 × 19 mm stent was fitted coaxially.

 After a further 3 months, he was referred to our Service of Vascular and Endovascular Surgery at Faculdade de Medicina de Botucatu, Botucatu, SP, Brazil, for endovascular repair of an infrarenal abdominal aortic aneurysm, which was achieved with no intercurrent complications and without significant increase in creatinine level 72h after the procedure. Intraoperative aortography showed 60% renal in-stent restenosis. He was regular taking three classes of antihypertensive medication plus sporadic use of clonidine, maintaining a mean systolic pressure of 150 mmHg, according to daily measurements taken at the health center. During follow-up, duplex mapping of the aorta showed that the aortic endoprosthesis was patent and free from leaks and that there was > 70% in-stent restenosis of the right renal artery (peak systolic velocity [PSV] = 475 cm/s, with a renal-aortic ratio [RAR] of 5.8). In view of this situation, the following treatment options were discussed:

1 - Kidney autotransplantation
2 - Conventional balloon angioplasty
3 - Angioplasty with placement of another intra-stent stent
4 - Angioplasty with a cutting balloon
5 - Angioplasty with placement of a DES
6 - Drug-coated balloon angioplasty

### Part II – What was done

 The patient was prescribed renal arteriography and treatment with a drug-coated balloon. Access was acquired via the left brachial artery and aortography identified 70% in-stent stenosis ( Figure 2 ). The right renal artery was selectively catheterized with a 5F 125 cm vertebral catheter, the lesion was crossed with a 0.035” 260 cm stiff hydrophilic guidewire, which was then changed for an extra-stiff 0.035” 260 cm guidewire, followed by progression of a 6F 90 cm introducer (Flexor – COOK®, Bloomington, USA), positioned in the vicinity of the emergence of the right renal artery ( Figure 3 ). A 4 × 40 mm balloon (Admiral Xtreme – Medtronic®, Minnesota, USA) was used to pre-dilate the lesion before definitive angioplasty was performed with 6 × 40 mm paclitaxel-covered balloon (Admiral In.pact – Medtronic®, Minnesota, USA), which was inflated to nominal pressure for 90 seconds. The immediate result was 30% residual stenosis, since a central “waist” remained, without significant recoil or other associated problems, and using approximately 40 mL of non-ionic low osmolality iodinated contrast ( Figure 4 ). 

**Figure 2:** Aortography via a brachial access, showing signs of intra-stent hyperplasia, confirming the duplex mapping findings.

**Figure 3:** Access sequence for intra-stent drug-coated balloon angioplasty. Selective catheterization of the right renal artery with a 5F 125 cm vertebral catheter, crossing the lesion with a 0.035” 260 cm stiff hydrophilic guidewire, swapping the guidewire for a 0.035” 260 cm extra-stiff, and advancing the 6F 90 cm introducer up to the emergence of the right renal artery.

**Figure 4:** Pre-dilation of the lesion with a 4 × 40 mm balloon (A) and angioplasty with a 6 × 40 mm paclitaxel-covered balloon, with signs of a “waist” in the balloon at the point of greatest stenosis, followed by complete inflation of the balloon up to its nominal pressure (B). Selective angiography for post-angioplasty control, showing residual stenosis of 30-40% (C).

 At 10 months, the patient exhibited clinical improvement, with blood pressure control confirmed by ambulatory blood pressure monitoring, mean systolic pressure of 130 mmHg, reductions in both classes and doses of antihypertensives (only enalapril 20 mg was maintained), and no abnormalities of creatinine and urea levels or of creatinine clearance. Follow-up to 24 months showed that these clinical improvements were maintained and all monthly duplex mapping examinations showed criteria for in-stent stenosis of 50-70% ( Figures 5 and 6 ), according to the criteria described by Chi et al. 13 (PSV from 225 to 315 cm/s and RAR from 3.12 to 4.66), up to 2014 ( Figure 7 ). 

**Figure 5:** Periodic assessments with renal duplex mapping. (A) Examination at 30 days after angioplasty; (B) Examination at 90 days after the procedure; (C) Examination at 6 months after the procedure; (D) Examination at 12 months after the procedure.

**Figure 6:** Curves for Doppler velocity measurement parameters over the first 9 months of post-angioplasty follow-up. Dotted lines indicate the 50-70% stenosis range, according to Doppler velocity measurement. Above, peak systolic velocity (PSV) in centimeters per second (cm/s). Below, renal-aortic ratio (RAR), obtained by dividing the PSV measured at the renal artery by the PSV at the suprarenal aorta.

**Figure 7:** Duplex mapping of renal artery, hilum, and parenchyma for control at 24 months post angioplasty with drug-coated balloon (DCB).

## DISCUSSION

 Atherosclerotic stenosis of the renal artery occurs in 1-5% of the hypertensive population 14 ^,^ 15 and has associations with abdominal aortic aneurysms in 20-38% of cases. 15 Despite the initial appeal to invasive treatment of atherosclerotic lesions of the renal arteries, questions are now asked about its true benefits in terms of preventing progression of chronic kidney disease and for real control of blood pressure levels. 16 ^-^ 19 In the case described here, the indication for angioplasty derived from the need for renal revascularization to treat atherosclerotic disease involving the only kidney. 20

 Restenosis after angioplasty is a limitation of the percutaneous revascularization technique, irrespective of whether stents are used. 16 ^-^ 21 A meta-analysis found a restenosis rate of 26% after balloon angioplasty and 17% after angioplasty with stents, 3 which supports the use of primary stenting (class I) for ostial stenosis of the renal artery, when the indications for endovascular intervention are respected. Restenosis rates after renal stent angioplasty with satisfactory initial results varied in the range of 6-40%, depending on the diameter of the vessel treated, the characteristics of the lesion, and the patient’s comorbidities. 2 ^,^ 3 ^,^ 8 Another possible reason for progression of the renal in-stent stenosis in the case described could have been the endovascular aortic aneurysm repair that the patient had undergone. 14 ^,^ 20 However, it is devices with transrenal attachment that can be related to progression of stenosis. In this case, the endoprosthesis used to repair the aneurysm is based on infrarenal attachment. 

 In-stent restenosis of the renal artery of the only kidney with preserved function is a severe complication in renal angioplasty and should be treated promptly. 12 There is no consensus on the best treatment for in-stent renal restenosis, but balloon angioplasty should be attempted first. 8 There are descriptions of use of cutting balloons, cryoplasty and fitting of a new stent within the first one, even though there is no good evidence for their use and they are not durable options for treatment of these lesions. There are also descriptions of treatment using drug-eluting stents; 8 ^,^ 22 ^,^ 23 but their use for in-stent restenoses has produced conflicting results and the superposition of metal meshes can cause excessive neointimal proliferation. 5 ^-^ 7

 In this context, drug-coated balloons are an interesting option. This technology can transfer antiproliferative drugs to the artery wall in a short period of time and does not require implantation of a carrier system. 10 The fact that the drug is rapidly brought into contact with the endothelial surface promotes faster vascular healing and reduces the localized inflammatory process. Surgeons’ familiarity with angioplasty balloons and the current availability of clinically safe and effective antiproliferative drugs enables their use with safety in a range of arterial territories. 10

 Paclitaxel is the antiproliferative agent that has been investigated in the greatest number of studies of drug-coated balloons. This drug is derived from the bark of a tree native to the Pacific (*Taxus brevifolia*), is rapidly absorbed through the cell membrane, because it is highly lipophilic, and is active by through bonding to the beta subunit of tubulin, which inhibits microtubule function. This causes structural modification of the cytoskeleton of smooth muscle cells, changing cellular proliferation and migration for approximately 14 days, without cytotoxicity or rebound effect. 10 ^,^ 24 In small-scale randomized clinical trials, paclitaxel-covered balloons reduced restenosis rates in patients with in-stent coronary stenosis and in femoropopliteal lesions. 24 ^,^ 25

 Despite observation of satisfactory results from use of DCBs in a range of territories, doubts remain with relation to widespread use. It is not known with certainty whether the technique is applicable to treatment of surfaces that have been manipulated previously, such as endarterectomy or intra-stent areas and the possibility of distal distribution of the drug or the impact this would have on visceral territories have not been investigated. 12 However, the low rates of complications related to these procedures suggest that they can be employed in cardiovascular interventions, with potential applications in the renal arteries, hemodialysis fistulae, venous territories, and even percutaneous valvoplasties. 9 ^,^ 10

 In the case described here, in view of the multiple manipulations the renal artery had already undergone, the necessity of the intervention, and the possibility of employing drug-covered balloons in the renal arteries and the intra-stent surface, we considered that using DCB could be a less invasive method to treat a complex situation. Over the course of short-term follow-up, the patient exhibited clinical benefits and his quality of life was improved by the treatment. The results achieved with DCBs in other territories and this isolated experience could contribute to suggesting that these devices have applications for renal in-stent restenosis.
